# Radiation-Induced Lung Injury—Current Perspectives and Management

**DOI:** 10.3390/clinpract11030056

**Published:** 2021-07-01

**Authors:** Mandeep Singh Rahi, Jay Parekh, Prachi Pednekar, Gaurav Parmar, Soniya Abraham, Samar Nasir, Rajamurugan Subramaniyam, Gini Priyadharshini Jeyashanmugaraja, Kulothungan Gunasekaran

**Affiliations:** 1 Division of Pulmonary Diseases and Critical Care, Yale-New Haven Health Bridgeport Hospital, 267 Grant Street, Bridgeport, CT 06610, USA; sunny.mandeep@gmail.com; 2 Department of Internal Medicine, Yale-New Haven Health Bridgeport Hospital, 267 Grant Street, Bridgeport, CT 06610, USA; jay.parekh@bpthosp.org (J.P.); prachi.pednekar@bpthosp.org (P.P.); soniya.abraham@bpthosp.org (S.A.); jgini2912@gmail.com (G.P.J.); 3 Department of Radiology, Yale-New Haven Health Bridgeport Hospital, 267 Grant Street, Bridgeport, CT 06610, USA; gaurav.parmar@bpthosp.org; 4 Department of Internal Medicine, University at Buffalo, 462 Grider Street, Buffalo, NY 14215, USA; samarnas@buffalo.edu; 5 Department of Pulmonary Critical Care Medicine, St. Louis University, 3635 Vista Ave, St. Louis, MO 63110, USA; rajamurugan.subramaniyam@health.slu.edu

**Keywords:** radiation, pneumonitis, radiation-induced lung injury

## Abstract

Radiotherapy plays an important role in the treatment of localized primary malignancies involving the chest wall or intrathoracic malignancies. Secondary effects of radiotherapy on the lung result in radiation-induced lung disease. The phases of lung injury from radiation range from acute pneumonitis to chronic pulmonary fibrosis. Radiation pneumonitis is a clinical diagnosis based on the history of radiation, imaging findings, and the presence of classic symptoms after exclusion of infection, pulmonary embolism, heart failure, drug-induced pneumonitis, and progression of the primary tumor. Computed tomography (CT) is the preferred imaging modality as it provides a better picture of parenchymal changes. Lung biopsy is rarely required for the diagnosis. Treatment is necessary only for symptomatic patients. Mild symptoms can be treated with inhaled steroids while subacute to moderate symptoms with impaired lung function require oral corticosteroids. Patients who do not tolerate or are refractory to steroids can be considered for treatment with immunosuppressive agents such as azathioprine and cyclosporine. Improvements in radiation technique, as well as early diagnosis and appropriate treatment with high-dose steroids, will lead to lower rates of pneumonitis and an overall good prognosis.

## 1. Introduction

Inflammation of lung tissue is generally referred to as pneumonitis. When incited by an infectious agent, the resulting acute lung inflammation is categorized as pneumonia. The term pneumonitis is frequently used with non-infectious pathological conditions involving chronic hypersensitivity, radiation, aspiration, and drug-induced inflammation of the lung [1].

Radiation-mediated lung injury was recognized after the discovery of X-rays in the early 1900 [2]. Lung, breast, esophagus, thymus, and hematologic malignancies are commonly treated with radiation of the thorax. Ionizing radiations damage the DNA and subsequently, through various mechanisms, results in the death of the exposed cell [3]. Ionizing radiation is toxic to both normal and tumor cells. Factors which determine the extent of toxicity are based on radiation beam characteristics, fractionation, the volume of normal tissues irradiated, radiosensitizers, and underlying preexisting connective tissue disorders [4]. The sensitivity of the lungs to radiation is the common denominator in determining the dose that can be delivered [4]. We aim to briefly describe the epidemiology, pathophysiology, risk factors, imaging characteristics of radiation induced lung injury (RILI) and review the evidence regarding management of RILI.

## 2. Epidemiology

The incidence of radiation injury and the subsequent pneumonitis directly correlates with the amount of radiation exposure. The radiation dose varies with organ and type of tumor. The incidence of radiation-mediated lung damage with lung tumors is high compared with breast or chest lymph node neoplasms [5,6].

Dose-volume analysis of pneumonitis incidence is found to be around 9.4% in non-small cell lung cancer (NSCLC) patients treated with stereotactic body radiation therapy [7]. Other tumors treated with radiation to the chest but not involving the lung parenchyma may have a lower propensity for radiation pneumonitis considering the lower amount of radiation received for those neoplasms [8]. Interestingly, the incidence increased from 1.1 to 14% when paclitaxel was used as a combination with radiotherapy for breast cancer. Moreover, sequential exposure increased the risk further [9].

## 3. Pathophysiology

Radiation breaks chemical bonds at the atomic level on the exposed tissues. Tissue damage is attributed to free radicals which are generated in tissue water. The generated free radicals damage the DNA of the cells, making it difficult for the normal cells and tumor cells to regenerate. The tumor cells do not possess the reparability of normal cells and are destined to die. Fractionated doses allow the normal cells to recuperate during the breaks. Fractionation is widely used to minimize damage to normal tissue. The common mechanisms of cell death involve mitotic cell death, apoptosis, autophagy, and necrotic cell death [5,10].

Genetic susceptibility is also responsible for increasing the risk of radiation pneumonitis in NSCLC patients treated with radiotherapy [11]. Post irradiation dysregulation of cytokine signal transduction results in elevated levels of IL-1, IL-6, TGF-beta, Platelet-derived growth factor (PDGF), proangiogenic hypoxia-inducible factor-1 alpha, vascular endothelial growth factor, and Interferon-gamma. The TGF-Beta level elevation was previously assumed to predict the risk of pneumonitis, whereas a recent meta-analysis identifies the evidence to be inadequate [12]. Type 1 pneumocytes are terminally differentiated and are often not affected by irradiation. In a rat model exposed to different doses of X-rays, within a few hours of irradiation the lungs became hyperpermeable due to damage to type II pneumocytes and developed increased surfactant production visible on electron microscopy, which would predispose to the risk of pneumonitis [13].

## 4. Predisposing Factors of Lung Injury

Many factors determine the degree of development of radiation-induced pneumonitis. We can divide them into treatment-related risk factors and patient-related risk factors. Table 1 summarizes the various predisposing risk factor for radiation-induced lung injury. Treatment-related risk factors include radiation total dose, fractionation, and dose rate, the volume of irradiated lung and portals, beam arrangement, and concurrent chemotherapy [8].

### 4.1. Treatment-Related Risk Factors

#### 4.1.1. Total Radiation Dose

The correlation between radiation pneumonitis and total radiation dose is not linear; instead, it increases significantly after a threshold dose is reached [14]. Lung damage is rarely seen with total doses less than 20 Gray (Gy); commonly seen with doses of 30 Gy to 40 Gy, and nearly always seen with a dose greater than 40 Gy [2,15].

#### 4.1.2. Fractionation and Dose Rate

By dividing the radiation doses, the biological impact of radiation is reduced. The daily dose fractions number and size have a direct impact on the risk of radiation pneumonitis [14]. A recent study showed that a daily dose fraction of more than 2.67 Gy had the most significant risk with the development of radiation of pneumonitis [16]. 

#### 4.1.3. The Volume of Irradiated Lung

The risk of radiation pneumonitis is proportional to the volume of lung irradiated. The usual total dose used for most cancers exceeds 50 Gy, which is far greater than the critical value. Hence the volume of the lung becomes the most important risk factor for radiation-induced pneumonitis [17]. The greater the lung volume irradiated higher the risk; with >50% volume irradiation, there is a greater prevalence of radiation pneumonitis [14].

#### 4.1.4. Technique of Irradiation

Different types of portals and beam angles are used for thoracic cancers. Radiation pneumonitis conforms to portals and beam arrangement. Conformal radiation therapy (CRT) aims to deliver high radiation to the shape of the target volume in three dimensions and minimize the dose delivery to normal tissue. More specialized CRT includes intensity-modulated radiation therapy (IMRT) and stereotactic body radiation therapy (SBRT). According to current published literature, newer techniques IMRT, SBRT, and proton beam therapy have reduced the incidence of clinically significant radiation pneumonitis as compared to conventional radiation therapy [18,19,20,21]. Several recent advances to minimize radiation dose to adjacent organs at risk have been developed and studied, for example, using 4D CT for functional avoidance planning, pre-treatment with FDG (imaging-based biomarkers), and normal tissue complication probability (NTCP) models for pre-treatment planning and selection of treatment technique. Studies have shown there is marked functional heterogeneity throughout the lung volume that may be exacerbated in the presence of underlying lung disease. Using 4D CT ventilation imaging to identify areas of high functioning lung tissue with an intent to avoid them in radiation planning has generated interest [22]. 

To predict the risk of radiation pneumonitis, pre-treatment with FDG is studied. The concept behind this is thought to be increased susceptibility of pre-treatment inflammation in the lung to radiation injury that would likely manifest as increased FDG uptake, thus allowing for quantitative assessment of radiation pneumonitis. Quantitative Analysis of Normal Tissue Effects in the Clinic (QUANTEC) initiative is one of the NTCP models which has been used as a predictor. It demonstrated a relationship between the mean dose to the lungs (MLD) and the risk of RP for NSCLC patients and provided a quantitative estimate of the dose-response relationship. NTCP model-based approach to predict toxicities can be advantageous in deciding which patients stand to benefit from a particular treatment modality [22,23]. 

#### 4.1.5. Chemotherapy

Several chemotherapeutic agents, used for induction or concurrently, such as doxorubicin, taxanes, dactinomycin, bleomycin, cyclophosphamide, vincristine, mitomycin, gemcitabine, recombinant interferon-alpha, and bevacizumab can increase the risk of developing radiation pneumonitis [24,25,26]. Also, some agents are known to cause lung toxicity but also predispose lungs to pneumonitis from radiation.

#### 4.1.6. Immunotherapy

Major advances have been made in cancer therapy by the development and use of immune checkpoint inhibitors (ICI). ICI-associated pneumonitis is now a well-known complication with rates as high as 19% in patients with non-small cell lung cancer (NSCLC) [27]. The exact pathophysiology is unclear, but preclinical evidence suggests that radiation could increase the effectiveness of ICI therapy through its immune-stimulating effects on the lung tissue. This may increase the effectiveness of ICI but also potentially increase toxicity and development of pneumonitis with ICI therapy [28,29]. The evidence for this is conflicting and limited to observational studies and case reports. The secondary analysis of the KEYNOTE-001 trial showed a higher risk of pulmonary toxicity in patients receiving pembrolizumab and prior radiotherapy when compared to those without prior radiotherapy (63% vs. 40%) [30]. Although, the PACIFIC study did not find an increased risk of grade 3–4 pneumonitis in patients treated with durvalumab versus placebo (3.4% vs. 2.6%) [31]. These studies did not assess the effects of concurrent radiotherapy as patients were treated sequentially with radiotherapy and ICI. Louvel et al., described two cases of patients treated with concurrent ICI, anti-PD 1 and anti-PDL1, along with SBRT, who developed RILI at five months and three months respectively from the time of receiving radiotherapy [32].

### 4.2. Patient-Related Risk Factors

- Age and sex: Radiation-induced lung injury can occur in patients of all ages without gender preference. Older patients have more co-morbidities, lower functional status, lower cardiopulmonary reserve than younger patients, which may increase the risk of RILI with age [33,34].
- Interstitial lung disease: The presence of baseline ILD is a significant risk factor for grade 4 and 5 radiation pneumonitis [35]. ILD itself causes parenchymal inflammation, which can predispose to radiation-induced lung injury (RILI). In a recent single-center experience, the rate of radiation pneumonitis after receiving SBRT was higher in patients with pre-existing ILD (20% versus 6%) [36]. Pre-existing ILD also increases the risk of mortality in patients with RILI [37].
- Smoking and COPD: Data are conflicting with retrospective studies indicating less symptomatic radiation pneumonitis in these patient populations than with normal lungs [38,39,40,41]. In fact, smoking may have a protective impact on the development of RILI when compared to non-smokers [42,43]. However, other retrospective studies indicate an increased risk of radiation-induced lung injury in patients with COPD, specifically pulmonary emphysema [33,44]. Patients with lung cancer have an increased prevalence of COPD, and further prospective research is needed to understand the impact of COPD in RILI.
- Tumor-related factors: Higher rates of organizing pneumonia have been observed in women with breast cancer treated with concurrent radiation and endocrine therapy [45]. Similarly, concurrent use of tamoxifen in women with breast cancer increases the frequency of pulmonary fibrosis [46]. Breast cancer and lung cancer involving the mid-lower lung zones are more associated with RILI [12,47]. Patients with higher tumor volume have a higher percentage of irradiated lung and, therefore, a higher risk of RILI [48,49].

## 5. Phases of Lung Injury

- Acute Phase

This phase occurs within the first few days of irradiation where pulmonary changes manifest in the background even though the patient is asymptomatic. This phase is characterized by acute inflammatory changes resulting in vascular congestion, accumulation of inflammatory cells, and apoptosis of type 1 pneumocytes and intrapulmonary edema [53]. Cytokines like Tumor Necrosing Factor-alpha (TNF-α), Interleukin 6 (IL-6), Interleukin-1 (IL-1), Basic fibroblastic growth factor (bFGF), Platelet-derived growth factor β (PDGF β) are released within two weeks after radiotherapy. Accelerated oxidative DNA damage, hypoxia, reduced lung perfusion, and expression of Transforming Growth Factor beta 1 (TGFβ-1) is seen 6–8 weeks post radiotherapy [54].

- Latent Phase

This phase is characterized by goblet cell recruitment and ciliary dysfunction. Tenacious secretions from the goblet cells may induce further inflammation around these tissues. Epithelial and endothelial degeneration is also seen in this phase [55].

- Exudative Phase

This phase begins 3–12 weeks after radiation exposure lasts until 12–16 weeks of irradiation. At this stage, the patients develop symptoms due to degenerative changes from the clogged mucinous glands and the destruction of the minor blood vessels. Alveolar collapse occurs from the detachment of epithelium and endothelium, which is followed by pulmonary capillary narrowing and microvascular thrombosis. The hyaline membrane is formed from the desquamated pneumocytes, and fibrin-rich exudate secreted from the alveoli [54,55].

- Intermediate Phase

This phase is characterized by the destruction of hyaline membranes and healing by primary and secondary intention. The fibroblast and cell signaling pathways work to restore tissue integrity. Fibroblasts migrate and proliferate in the alveolar walls, which convert to myofibroblasts which leads to fibrosis. Fibrosis causes hypoxia which in turn leads to the release of pro-angiogenic and pro-fibrogenic factors resulting in chronic lung disease [8,56].

- Resolution Phase

This phase is characterized by the resolution of symptoms as the body’s innate immune mechanism clears the destroyed hyaline membranes and promotes healing by primary and secondary intention. The fibroblast and cell signaling pathways work to restore tissue integrity [5].

- Fibrosis

The healing process triggers fibroblast activation resulting in fibrosis. The trigger mechanism for fibrosis is largely unknown. This phase starts with 6–8 months of irradiation and continues to progress for many years. Some patients may experience dyspnea with right heart failure without any symptoms of radiation pneumonitis [5,6,8,17]. 

## 6. Diagnosis of Radiation Pneumonitis

### 6.1. Examination Findings 

The symptoms of malaise, non-productive cough, low-grade fever, dyspnea, and chest pain can occur with varying grades. Most symptoms are commonly noticed in the first 3–12 weeks after treatment, even though symptoms may occur within the first year of irradiation. Since symptoms are not specific, the other causes of infection, including cardiogenic causes, pulmonary embolus, and drug-related toxicity, etc., must be ruled out. Crackles on auscultation and other instances of normal vesicular breath sound frequently confound the physical examination. Hyperpigmentation/skin erythema are nonspecific and do not predict the risk of pneumonitis [6,57,58].

### 6.2. Laboratory Findings 

Type 2 pneumocytes synthesize higher levels of Serum Krebs von den Lungen-6 (KL-6) and serum Surfactant protein-D (SP-D) after irradiation. Their levels were elevated in interstitial pneumonitis and irradiation. Routine laboratory examination to rule out other causes is still necessary [35,59,60].

Clinical grading of pneumonitis has not been established, but a toxicity criterion for adverse events is devised by the National Cancer Institute, as illustrated in Table 2.

### 6.3. Complications

Radiation-induced lung injury follows an indolent course. Any acute decompensation should prompt a workup for infection, heart failure, and thromboembolic diseases. Few case reports of spontaneous pneumothorax following thoracic radiation for non-small cell lung cancer and lymphoma have been reported [64]. Radiation-induced organizing pneumonia has been reported after breast irradiation. Characteristic imaging features are infiltrates often migratory outside the radiation field, halo sign, persistent cough with or without shortness of breath [65]. Pulmonary necrosis causing cavitation, although exceedingly rare, has been reported in the late acute phase [66,67]. The infectious cause should be considered in the presence of a cavitary lesion in patients with prior thoracic radiotherapy [68].

### 6.4. Imaging

#### 6.4.1. X-rays

**Figure 1:**
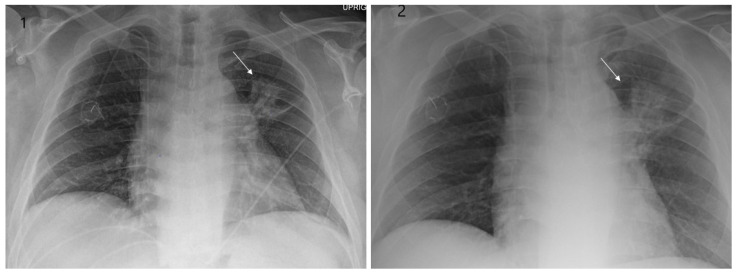
Chest X-ray showing radiation pneumonitis Image 1—Frontal chest X-ray showing left upper lobe mass (arrow), the patient also had a right internal jugular port placed. Image 2—Post radiation treatment frontal chest X-ray showing increasing alveolar and interstitial opacities in the left upper lobe and in the left lower lobe in a patient suspected of radiation pneumonitis.

Even though X-ray is non-specific to pneumonitis, it is usually the first investigation performed. During the early phases, the most common finding on chest radiograph is perivascular haziness which frequently progresses to alveolar opacities [39]. Chest radiographs can show ground-glass opacity and/or consolidation in the radiation port. Consolidation usually has a nodular appearance but can be more confluent/lobar along with the irradiated port. Findings can be seen outside the radiation port as well. Some uncommon findings are ipsilateral pleural effusion with or without adjacent atelectasis [66]. Chest radiograph can also show bilateral interstitial infiltrates mimicking heart failure or acute respiratory distress syndrome (ARDS) [69,70]. All irradiated patients usually have some degree of abnormalities in the X-rays. Some fail to show any radiographic evidence of lung injury with pneumonitis. Early features exhibiting mild opacification of vascular markings are common, with later stages showing dense opacities. A radiographic straight-line effect may indicate the direction of the radiation port along the lines of pneumonitis [39,71] (Figure 1).

#### 6.4.2. Chest CT-Scan

In the event of worsening symptoms after empirical antibiotics, Chest CT may provide more insights. Interestingly, the opacification lines in both X-ray and CT conform to radiation port rather than anatomical lines of lung structure, which could be diagnostic. Identified progression outside the lung field might suggest immune-mediated lymphocytic alveolitis [72]. Various stages of presentation provide different imaging outlooks (Table 3).

During the acute exudative stage, features of ground-glass attenuation or homogeneous consolidation may be noticed. A patchy consolidation that confirms the irradiation portal is also suggestive of the early phase. A discrete consolidation that conforms to the shape of the irradiation portal is proliferative changes of irradiation [71].

A chronic fibrosis stage with features of parenchymal distortion, traction bronchiectasis, and pleural thickening resulting in volume loss and irreversible changes are noticed. Refer to Table 2.

#### 6.4.3. Differential Diagnosis

Radiation-induced lung injury shares a magnitude of radiographic findings with other diseases affecting the lungs. In the current era of the coronavirus disease 2019 pandemic, it is crucial to distinguish from SARS-CoV-2 interstitial pneumonia as many clinical and imaging features overlap [73]. The most common difference would be an infection like viral pneumonitis, bacterial pneumonia (atypical or mycobacterial), or fungal pneumonia. This group of patients is at a higher risk of infections given their immunosuppressed status due to concurrent or sequential chemotherapy and immunotherapy. Other differences include organizing pneumonia, drug-induced pneumonitis (immune checkpoint inhibitor pneumonitis), e-cigarette or vaping-use associated lung injury (EVALI), thromboembolic disease (SARS-CoV-2 infection also increases the risk of venous thromboembolism), interstitial lung disease, heart failure, lymphangitic carcinomatosis, and progression of malignancy [66,74,75,76,77,78,79,80].

#### 6.4.4. Newer Imaging Techniques

Artificial intelligence or machine-based learning algorithms have been evaluated recently for lung nodule and coronary artery calcium detection [81,82]. Giordano et al. studied the performance of a machine-based learning algorithm in distinguishing radiation pneumonitis from COVID-19 pneumonia. The algorithm was able to distinguish radiation pneumonitis from COVID-19 pneumonia with a sensitivity of 76% and specificity of 63% when COVID-19 risk probability was set at 30% [73]. Although, in another study by Mallio et al., a similar deep learning algorithm was unable to distinguish COVID-19 pneumonia and ICI treatment-related pneumonitis [83]. Recent studies have evaluated the role of pre-treatment 18F-2-fluoro-2-deoxyglucose positron emission tomography (FDG-PET) on predicting the risk of radiation-induced lung injury. These scans are often performed in patients with malignancy and can be incorporated in predicting the risk of RILI without additional testing burden on the patient. The 95th percentile of the standard uptake value (SUV_95_) was found to be predictive of RILI in on retrospective study (*p* = 0.016) [22,84].

#### 6.4.5. Pulmonary Function Tests

Even though PFTs do not directly diagnose radiation pneumonitis, a pre-existing obstructive lung condition with significant decreased pulmonary function test pre/post-irradiation can help differentiate exacerbation from Pneumonitis [85]. 

As the lungs are impaired post-irradiation, the diffusion capacity of the Carbon monoxide reveals a reduced DLCO. Higher reductions in DLCO before and after irradiation might predict the development of Pneumonitis [86].

#### 6.4.6. Bronchoscopy 

The lymphocyte count is generally elevated with inflammatory conditions; an irradiated lung shows elevated activated neutrophils, eosinophils, and macrophages. It is not necessarily performed to diagnose RP but to rule out other causes like infection, bleeding, or local spread of the tumor cells [13].

## 7. Management of Radiation Pneumonitis

### 7.1. Treatment Strategies

#### 7.1.1. Minimally Symptomatic Patients

Some minimally symptomatic patients are spontaneously relieved of their symptoms. Intervention is warranted in minimally symptomatic patients if there is a loss of function of over 10% on PFT. Follow-up with the assessment of symptoms, chest radiographs, and pulmonary function tests are necessary. A short course of 14 days Budesonide inhaler 800 micrograms twice a day has been proved beneficial [87,88]. Most of the patients with mild symptoms require inhaled steroids for several months.

#### 7.1.2. Symptomatic Patients with Subacute Radiation Pneumonitis

Dyspnea that interferes with activities of daily living and impaired respiratory function would benefit from oral corticosteroids. Prednisone (up to 60 mg/day) is administered for 2–4 weeks and tapered over 3–12 weeks gradually [89,90,91,92,93,94]. A relapse of the symptoms during the taper would require stepping up the dose. When prednisone is continued for over a month, pneumocystis pneumonia prophylaxis is warranted [90]. Some patients are resistant to steroids exhibiting elevated KL-6 protein levels. Such patients can benefit from Azathioprine or Cyclosporine A [91].

#### 7.1.3. Organizing Pneumonia

Cryptogenic organizing pneumonia protocols may be generally followed in patients with minimal symptoms. Periodic assessment for worsening symptoms would suffice. In patients with evidence of respiratory impairment, oral glucocorticoid therapy (up to 1 mg/kilogram body weight) can be administered for 4–8 weeks. Tapered dose reduction can be continued until all the symptoms are relieved [95].

#### 7.1.4. Pulmonary Fibrosis

A high dose of fractionated radiation may result in fibrosis. Lung fibrosis usually does not respond to glucocorticoids and avoiding them at this stage could prevent glucocorticoid-related side effects. Appropriate supportive therapy can help in symptomatic patients [3].

### 7.2. Treatment Options

#### 7.2.1. Steroids

One of the original studies investigating the use of steroids in radiation-induced lung disease dates back to 1953 [59]. In 1993, an animal model study explored histopathological changes in radiation-induced pneumonitis in mice and alterations in the same with the administration of steroids. The researchers found that steroids were beneficial in limiting the initial interstitial edema, protein leak, and alveolitis due to radiation injury. However, they did not see any change in the natural progression of the disease to fibrosis [93].

In 2006, the first retrospective analysis was which looked at the use of corticosteroids in radiation-induced lung injury was published. The selected population was exposed to 50 to 70 Gy of thoracic radiotherapy. Interestingly, in the study, about 80% of the patients who developed radiation pneumonitis showed no progression of the disease despite not using steroids. The radiographic progression of the disease appeared to be faster in the group receiving steroids. Out of the patients who received steroids, the symptomatic benefit was seen in about 90%; however, it did not appear to be sustained. The study used a dose of 30 to 40 mg of prednisone. Since it was a retrospective study, it appears based on the results that patients exposed to steroid use were more likely to have severe disease and would be difficult to interpret the benefit of steroid use in this setting [94].

A 2013 study compared spirometry values in radiation pneumonitis groups stratified based on steroid use and found no evidence to suggest steroids improve the pulmonary function test profile among radiation pneumonitis patients [96]. In 2016, a small single-center study involving 24 patients with grade 2 pneumonitis showed some symptomatic benefit with the use of budesonide 800 ug twice a day use in the inhaled form. Since then, it has become the mainstay of treatment for clinically symptomatic grade 2 pneumonitis [87]. Besides this study, there have been few sporadic case reports regarding clinical benefit from inhaled corticosteroids in the setting of early radiation pneumonitis, but strong conclusive data has been lacking due to the absence of large, randomized trials [88,97].

Overall, current evidence demonstrating significant long-term benefit with steroid use in radiation pneumonitis is lacking as there are no placebo controlled human trials showing alteration of the natural progression of the disease. Further studies in this aspect would be particularly beneficial since steroids seemingly remain the mainstay of therapy in established disease with a progressive course [8].

#### 7.2.2. ACE Inhibitor

The beneficial effect of ace inhibitors in mitigating radiation pneumonitis was first reported in a study conducted in the late 20th century by Ward and colleagues, which has been redemonstrated in numerous animal model-based studies. In this experimental study, rats were exposed to radiation and subsequently monitored for four indices of pulmonary endothelial dysfunction, including ACE. They found a dose dependent correlation in the reduction of ACE activity with radiation. Subsequently, in the study, they found that radiation-induced suppression of ACE activity is mitigated by captopril [98,99,100].

Subsequently, in 2000, a study was published which included 200 patients who were looked at retrospectively. In this study, patients were stratified based on ACE inhibitor use, and although only 26% percent of the patients were using the ACE inhibitor, it showed no difference in the incidence of pneumonitis among both the groups, thus questioning the benefit of ACE inhibitors in mitigating radiation-induced lung injury in human subjects [101].

In 2009, Ghosh and colleagues conducted a study on rats in which they looked at breathing rates as a marker of lung damage due to radiation and compared the same in groups of rats on losartan and captopril. They found that radiation-induced damage was mitigated in rats even if the drug was started a week after the radiation. They also concluded that survival was improved with captopril and found a statistically significant reduction in breathing rate among rats on captopril [102].

In 2011 a retrospective analysis was conducted on over 150 patients for more than a decade who underwent high dose radiation for non-small cell lung cancer. This study redemonstrated the beneficial effect of ACE inhibitors in radiation pneumonitis. The study found that there was an inverse correlation between ACE inhibitor used for other conditions and radiologic evidence of radiation-induced lung damage, which remained significant despite multivariate analysis. The study also found a non-statistically significant reduction in survival from 18 to 15 months [103,104]. In the early 2000s, a retrospective study was conducted among non-stage 4 lung cancer patients. Among 160 patients that were included in the study, close to 40 percent of the patients were on the ACE inhibitor. This study found a statistically significant reduced incidence of grade 2 or higher RP from 11% to 2% among patients on ACE inhibitors vs. those not on it [52].

In 2012, an experimental study looked at the use of ACE inhibitors from a different perspective. Researchers found histological evidence of a reduction in lung fibrosis among rats who were on ace inhibitors redemonstrating the possibility of the role of ACE in hydroxyproline synthesis. In this study, rats were exposed to thoracic radiation and then were studied to look for long term effects of radiation in the form of lung collagen synthesis. The rats that survived initial radiation pneumonitis after the first six weeks subsequently showed increased collagen in the lung compared to control rats not exposed to radiation, hinting towards the possibility of increased collagen synthesis and subsequent fibrosis as a separate entity rather than a progression of initial pneumonitis. They also found that this increase in collagen synthesis was not present in rats exposed to ace inhibitors [105]. This mitigation of lung collagen with ace inhibitor was redemonstrated by another study later [106].

Subsequently, over the last decade, several retrospective studies demonstrated a decreased incidence of radiation pneumonitis among patients receiving SBRT on ace inhibitors. However, there has been no definitive evidence to show its benefit in terms of long-term outcomes [107,108,109].

A pilot, double-blind, placebo-controlled, randomized trial was conducted with Lisinopril 20 mg among patients receiving curative radiotherapy. The study was limited by a low accrual rate but among the 23 patients who were randomized, patients receiving lisinopril had less cough, less shortness of breath, fewer symptoms from lung cancer, less dyspnea with both walking and climbing stairs, and better overall quality of life demonstrating the benefit of ace inhibitors in reducing symptoms of radiation-induced lung injury [110].

Overall, we conclude that there is evidence of benefit with ace inhibitors among patients undergoing exposure to radiation in terms of mitigation of radiation-induced suppression in ACE activity. It appears this benefit correlates clinically in patients with reduced incidence of symptomatic radiation pneumonitis. There seems to be some interest regarding the potential of ACE inhibitors to limit lung fibrosis; however, this is not conclusive. Again, there is a lack of strong evidence to conclude the effects of ACE inhibitors on long term outcomes and mortality among patients who develop radiation pneumonitis and is a potential area for new studies [111,112].

#### 7.2.3. Amifostine

Amifostine, an organic thiophosphate, has been studied in lung cancer for its cytoprotective effects against anti-tumor therapies. One of the studies looking at amifostine was conducted in rat models receiving radiation therapy for lung cancer. In this study conducted in 2009, amifostine showed a benefit in reducing TGF-beta levels and significantly reducing the respiratory rate in rats receiving radiation therapy, postulating the possibility of the potential benefit of amifostine in mitigating cytokine mediated toxicity from radiation [113].

A randomized trial conducted in Greece in 2001 showed a statistically significant reduction in the incidence of grade 2 pneumonitis in amifostine plus radiotherapy arm compared to radiotherapy alone arm paving the way for a clinically protective benefit with amifostine against radiation pneumonitis [114]. Similar results were redemonstrated in a 2002 study in non-operable NSCLC with a statistically significant reduction in the incidence of pneumonitis with amifostine, however, the use of amifostine was not associated with any significant survival benefit [115].

Amifostine is not only showing promising results in reducing incidence but is also ameliorating the effect of radiation pneumonitis on PFTs [116]. 

In 2006, a 14 randomized controlled trial based metanalysis consisting of 1400 patients consolidated evidence to demonstrate a significant benefit with the use of amifostine for radiation-induced injury not only to the lungs but also for radiation-induced xerostomia and esophagitis [117]. 

Overall, there is growing data for benefits with amifostine in radiation pneumonitis, which can be consolidated in the years to come for consideration of the standard of practice (Table 4).

## 8. Radiation Pneumonitis and Mortality and Outcomes

The data for survival and outcome in patients with radiation pneumonitis is influenced by concurrent malignancy. A retrospective study published in 2001 evaluated records of about 250 patients who underwent radiotherapy and found that about half of the patients developed radiation pneumonitis. 

RP was mild in 69 (36%) and severe in 25 (13%) patients. The 3-year survival rates of the patients who experienced no, mild, and severe RP were 33.4%, 38.2%, and 0%, respectively. Interestingly, the survival rate of the patients who experienced severe RP was significantly poorer than the patients in the other group suggesting poor outcomes with severe radiation pneumonitis. They also found no survival difference with steroid use [118]. 

In 2002, a small study was conducted in patients with severe radiation pneumonitis who were treated with radiotherapy for non-small cell lung cancer. In this study, the mortality rate was about 50 percent among patients within two months of the onset of radiation pneumonitis. Subsequently, on multivariate analysis, the extent of radiation-induced lung injury correlated with poor outcomes [119].

Another large Sweden based retrospective study looked at adverse effects of radiation among patient’s lung cancer and found no difference in the Kaplan Meier curve among patients who developed radiation pneumonitis [120]. Another study identified roughly 1700 patients who underwent curative radiotherapy and screened for patients who developed fatal radiation pneumonitis. The median survival time following pneumonia symptom appearance was 53 days. The 6- and 12-month overall survival rates were 34.8% and 13.0%, respectively [121].

A multivariate analysis on 100 patients with NSCLC who underwent radiotherapy found that the development of radiation pneumonitis was associated with reduced survival among patients from 29 months to 8 months [122].

Overall, it appears that survival is dependent on the severity of radiation pneumonitis and malignancy characteristics. Meanwhile, there was some evidence that radiation pneumonitis does affect the quality of life based on patient-reported scores [123].

## 9. Conclusions

Radiation-induced lung injury is a well-known complication of radiotherapy. With the increasing use of radiotherapy for thoracic cancers, there is an increasing risk of RILI. With the advent and increasing use of immune checkpoint inhibitors, it is becoming difficult to distinguish pneumonitis induced by ICI from radiation pneumonitis. Therefore, it is now more important than ever to maintain a high index of suspicion for this complication. Predisposing risk factors should be evaluated before initiating radiotherapy. CT chest remains the most readily available imaging modality, but recent advances in machine-based learning and PET-CT scans might change this in the future. In the current era, SARS-CoV-2 infection and e-cigarette or vaping-use associated lung injury (EVALI) should be included in the differential diagnosis. More common differentials like infection, thromboembolic disease, and heart failure should be excluded. Multidisciplinary discussions involving pulmonologists, radiation oncologists, and thoracic radiologists might be helpful in reaching a consensus diagnosis. Evidence on management is limited to inhaled and systemic corticosteroids at this time. Further research is needed to address this evolving clinical condition.

## Figures and Tables

**Table 1:** Predisposing risk factors for radiation-induced lung injury.

Risk Factors	Type	Remarks
Treatment-related risk factors	Total radtion dose	Commonly seen with doses greater than 40 Gy. Higher the mean lung dose, greater the risk and severity of RILI [50]. Higher dose fraction, Volume of lung receiving at least 20 Gy > 10% (V_20_ > 10%) and mean lung dose > 6 Gy are associated with higher grade RILI [51].
	Fractionation and dose rate	
	Irriated lung volume	
	Irradiation technique	Newer radiation delivery techniques IMRT, SBRT, and proton beam therapy have reduced the incidence of clinically significant RILI.
	Chemothreapy	Induction and concurrent chemotherapy increases the risk of RILI.
	Immunotherapy	Immune checkpoint inhibitor (ICI) therapy, concurrently or sequentialy increases the risk of RILI.
Patient-related risk factors	Age and sex	RILI should be considered in patients of all ages and sex. Higher the age is likely associated with greater risk of RILI. In one retrospective review grade II or higher RILI was significantly increased in patients >70 years of age [52].
	Smoking status	Smoking may have protective impact in development of RILI.
	Pre-existing lung disease like COPD, ILD	Data regarding impact of COPD is conflicting, some reports indicating increased risk of RILI. ILD is a significant risk factor for development of RILI and is associated with increased mortality.
	Tumor type, location and size	Concurrent endocrine therapy in women with breast cancer have increased risk of RILI. Higher tumor volume and mid-lower lung zone location.

**Table 2:** Common Terminology Criteria for Adverse Events version 5.0 for pneumonitis [61,62,63].

Grade	Incidence	Clinical	Radiologic
1	20–24%	Asymptomatic to minimally symptomatic	ground glass opacities, less than 25% of lung involvement
2	18–22%	symptomatic requiring treatment, limitation of ADLs, but no oxygen requirement	extensive ground glass opacities extending beyond therapy field with signs of no to minimal focal consolidation, involvement of 25 to 50% of lung
3	8–16%	symptoms with oxygen requirement	clear evidence of focal consolidation with or without evidence of fibrosis, more than 50% involvement
4	2–4%	severe symptoms with persistent oxygen requirement or assisted ventilation	dense consolidations, atelectasis, traction bronchiectasis with significant pulmonary volume loss

**Table 3:** Computed Tomography (CT) chest illustrating post-radiation therapy changes in the lungs.

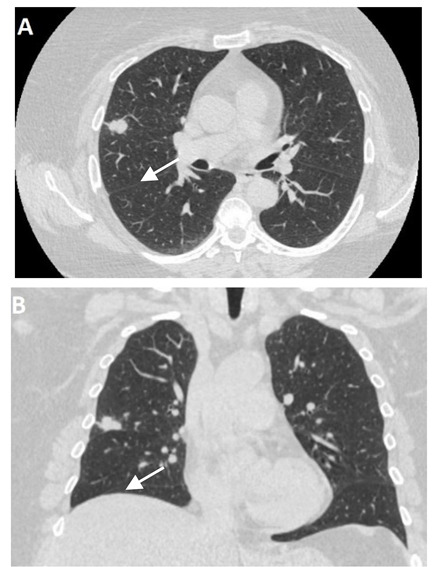	CT chest axial (image A) and coronal (image B) showing well-defined 2 cm nodular lesion in the Right Middle Lobe (RML) denoted by an arrow.
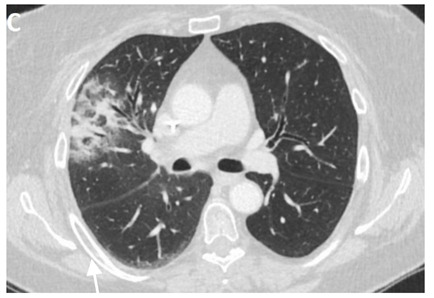	Post radiation therapy axial CT chest (image C), 1.5 months later showing ground-glass opacity and patchy airspace consolidation in RML conforming to the area of radiation field denoted by an arrow.
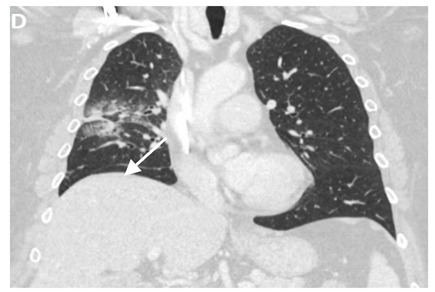	Same patient post-radiation therapy coronal CT chest (image D), 1.5 months later showing ground-glass opacity and patchy airspace consolidation in RML conforming to the area of radiation field denoted by an arrow.
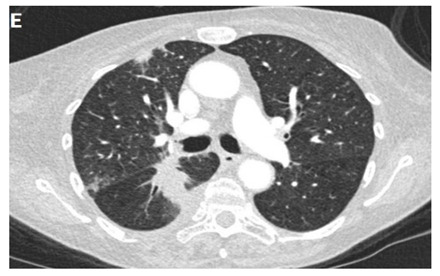	Axial CT chest (image E) showing irregular 5.5 cm mass lesion with spiculated margins in the Right Lower Lobe (RLL) with the background of emphysematous lungs denoted by an arrow.
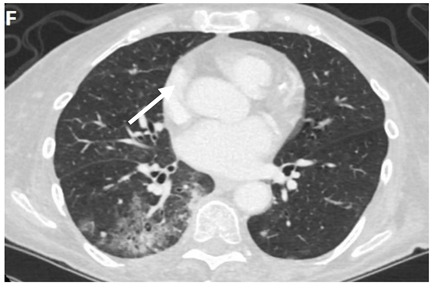	Axial CT chest (image F) showing predominantly ground-glass opacity and patchy focal consolidation in the RLL conforming to the area of radiation field denoted by an arrow.
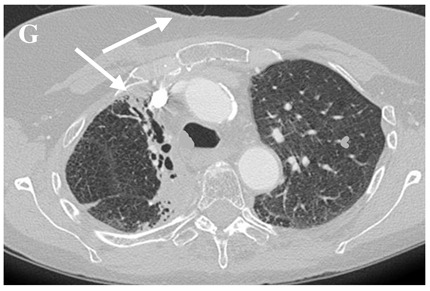	Axial CT chest (image G) showing dense fibrosis and bronchiectasis in the right upper lobe (RUL) post radiotherapy denoted by an arrow.

**Table 4:** Important studies evaluating various therapeutic options for radiation-induced lung injury.

Author (Year), Study Drug	Total Patients (N)	Methods	Results	Remarks
Henkenberens et al. (2016), corticosteroids [87]	24	24 patients with NSCLC received radiation therapy and developed grade II RILI were treated with high dose inhaled corticosteroid (Budesonide 800 mcg twice daily) for 14 days followed by oral prednisolone (0.5 mg/kg bodyweight, at least 50 mg/day). Median follow up 18 months.	18 patients showed significant symptomatic patients after ICS treatment. 6 patients who did not respond to ICS, had significant clinical improvement with oral prednisolone.	16/18 responders to ICS did not have underlying COPD and were treated for a median of 7.7 months with ICS
Kharofa et al. (2012), angiotensin-converting enzyme (ACE) inhibitor [52]	162	Retrospective study, 162 patients with NSCLC treated with radiation therapy were included. The use of ACE inhibitors, steroids, statins were assessed for relationship with grade II RILI or higher.	64% patients had grade III disease. ACE inhibitor users had significantly lower rates of grade II or higher RILI (2% vs. 11%, *p* = 0.032).	38% patients were ACE inhibitor users. V_20_ ≤ 37% and mean lung dose ≤ 20 Gy.
Sio et al. (2019), angiotensin-converting enzyme (ACE) inhibitor [110]	23	Double-blinded, placebo controlled randomized controlled trial (RCT) of patients receiving radiation therapy assigned to 20 mg lisinopril daily or the placebo group. Multiple patient related outcome surveys used to evaluate the primary endpoint.	12 patients received lisinopril and 11 received placebo. Patients in the treatment arm had less cough, shortness of breath on exertion and fewer symptoms of lung cancer (*p* < 0.05)	Accrual was less than expected. All patients received concurrent chemotherapy.
Sasse et al. (2006), Amifostine [117]	1451	Meta-analysis of 15 RCT comparing the use of amifostine plus radiotherapy with radiotherapy alone.	Lower odds of acute pneumonitis in the amifostine group (OR, 0.15; CI, 0.07–0.31; *p* < 0.00001)	Amifostine also significantly reduced the risk of developing mucositis, esophagitis, xerostomia and dysphagia.

## Abbreviations

RILI	Radiotherapy induced lung injury
RP	Radiation pneumonitis
RF	Radiation fibrosis
RT	Radiotherapy
SBRT	Stereotactic body radiation therapy
ICI	Immune checkpoint inhibitor
NSCLC	Non small cell lung cancer
